# Overfeeding during a critical postnatal period exacerbates hypothalamic-pituitary-adrenal axis responses to immune challenge: a role for adrenal melanocortin 2 receptors

**DOI:** 10.1038/srep21097

**Published:** 2016-02-12

**Authors:** Guohui Cai, Ilvana Ziko, Joanne Barwood, Alita Soch, Luba Sominsky, Juan C. Molero, Sarah J. Spencer

**Affiliations:** 1 School of Health and Biomedical Sciences, RMIT University, Melbourne, Vic., Australia

## Abstract

Early life diet can critically program hypothalamic-pituitary-adrenal (HPA) axis function. We have previously shown rats that are overfed as neonates have exacerbated pro-inflammatory responses to immune challenge with lipopolysaccharide (LPS), in part by altering HPA axis responses, but how this occurs is unknown. Here we examined neonatal overfeeding-induced changes in gene expression in each step of the HPA axis. We saw no differences in glucocorticoid or mineralocorticoid receptor expression in key regions responsible for glucocorticoid negative feedback to the brain and no differences in expression of key HPA axis regulatory genes in the paraventricular nucleus of the hypothalamus or pituitary. On the other hand, expression of the adrenal melanocortin 2 receptor (MC2R) is elevated after LPS in control rats, but significantly less so in the neonatally overfed. The *in vitro* adrenal response to ACTH is also dampened in these rats, while the *in vivo* response to ACTH does not resolve as efficiently as it does in controls. These data suggest neonatal diet affects the efficiency of the adrenally-mediated response to LPS, potentially influencing how neonatally overfed rats combat bacterial infection.

Obesity is characterised by a chronic low-grade inflammatory profile that contributes to leptin resistance and the onset of diabetic symptoms^1^. This altered inflammatory profile can also lead to an increased susceptibility to and morbidity and mortality from infections^2^. For instance, obese patients are twice as likely to develop pneumonia, up to six times as likely to contract a post-surgical infection, and 2.1 times as likely to die in intensive care due to infection-related complications than normal-weight patients are^2^,^3^. Our recent findings suggest the hypothalamic-pituitary-adrenal (HPA) axis may play an important role in regulating this susceptibility^4^.

We have recently demonstrated, in rodents, an overweight/obese phenotype that occurs as a result of overfeeding in early life leads to a markedly exacerbated neuroimmune response to the bacterial mimetic, lipopolysaccharide (LPS)^4^. Neonatally overfed rats have increased white adipose tissue expression of pathogen-associated molecular pattern recognition receptor toll like receptors (TLR)4 and 2. They have increased phosphorylation of inhibitory factor κB, elevated circulating pro-inflammatory cytokine levels, and higher fevers after LPS compared with rats from a control background. This response is likely to be at least partly due to macrophage proliferation within adipocytes and a subsequent elevated expression of TLR4 leading to enhanced downstream effects of the LPS in these rats. However, findings from this study also suggest these neonatally overfed rats have important changes in their HPA axis responses to LPS^4^,^5^.

The HPA axis is the body’s principal endocrine response to stress, including to immune challenge^6^,^7^. HPA axis activation culminates in glucocorticoid (GC) release into circulation, which acts at immune cells to interfere with nuclear factor κB-mediated transcription of pro-inflammatory cytokines, thus attenuating fever^8^. We have seen neuronal activation in the paraventricular nucleus of the hypothalamus (PVN; as measured by immunohistochemistry for Fos) is markedly increased after LPS in neonatally overfed rats compared with controls, as was anticipated based on the pro-inflammatory cytokine and febrile responses^4^,^5^. Interestingly, however, circulating corticosterone concentrations are not simply higher after LPS in the obese. Neonatal overfeeding is associated with a slower corticosterone response so that the peak is reached significantly later after exposure to LPS than in controls^4^. These findings imply an inefficiency in HPA axis negative feedback may contribute to an exacerbated response to an immune challenge.

The HPA axis is remarkably sensitive to early life programming events. For instance, high intensity parenting (high levels of licking and grooming accompanied by significant arched-back nursing) in the rat leads to long-term increases in glucocorticoid receptor (GR) gene expression in the hippocampus resulting in attenuated HPA axis responses to stress^9^,^10^,^11^,^12^. In this line, humans with a history of abuse during childhood have reduced hippocampal GR gene expression and exacerbated responses to stress throughout life, as well as increased susceptibility to illness; effects that are mediated through epigenetic mechanisms^12^,^13^,^14^,^15^. The HPA axis is also influenced by early life immune experience with neonatal immune challenge leading to an exacerbated HPA axis responses to an immune challenge of the same type despite a suppression of the febrile and sickness behaviour components of the response^16^,^17^. In this case, the mechanism likely involves hypersensitivity / hyper-expression of liver and spleen TLRs leading to early activation of the HPA axis through an early liver-derived increase in prostaglandins^16^.

In the present investigation, we aimed to examine changes in HPA axis function in male rats made obese due to neonatal overfeeding to determine if alterations in GC sensitivity or negative feedback are involved in the exacerbated response to LPS seen in these animals. Here we induced neonatal overfeeding by manipulating the litter sizes in which the rat pups are suckled, generating litters of 4 (small litter; SL; neonatal overfeeding) and 12 (control litter; CL). When the rats reached adulthood, we examined changes in neuronal activation and gene expression in several steps of the HPA axis that were likely to be influenced by neonatal overfeeding.

## Results

### Neonatal overfeeding enhances short- and long-term weight gain

**Figure 1:**
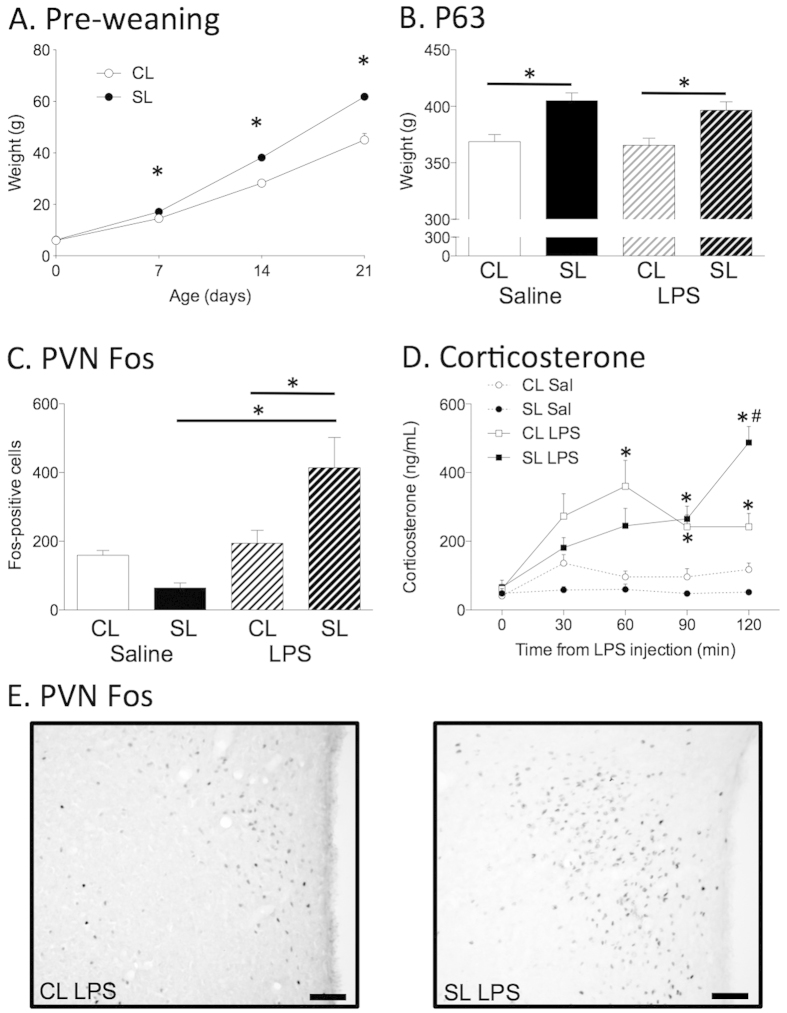
(**A**) Pre-weaning and (**B**) Adult (postnatal day (P)63) body weights of rats raised in control (CL) and small (SL) litters. (**C**) Paraventricular nucleus of the hypothalamus (PVN) neuronal activation (Fos) in response to i.p. LPS. (**D**) Plasma corticosterone in response to i.p. LPS. (**E**) Photomicrographs of the PVN from representative CL and SL LPS-treated rats. Scale = 100 μm. Data are mean + SEM. *compared with saline-treated group for the same litter size, ^#^compared with CL-LPS; *p* < 0.05.

As we have previously seen^18^,^19^,^20^, neonatal overfeeding (SL) is associated with accelerated growth during the suckling period (F_(3,66)_ = 30.46, *p* < 0.001) such that SL were significantly heavier than CL by postnatal day (P)7 and this was maintained through to P14 and P21 (n = 8 litters each; Fig. 1A). This weight phenotype was maintained into adulthood (F_(1,90)_ = 23.30, *p* < 0.001) and the groups allocated to saline or LPS treatments were not different from one another (Fig. 1B).

### Neonatal overfeeding exacerbates HPA axis responses to LPS

We have previously reported that male rats raised in SL have more neurons activated in the PVN in response to a single injection of LPS in adulthood than those raised in CL, as assessed by numbers of Fos-immunoreactive cells in the region^4^,^5^. They also have differences in their GC response to the LPS^4^. We replicate those findings here in that LPS led to significant activation of the PVN in SL but not CL rats and there were significantly more Fos-positive cells in the PVN in SL than CL after LPS (significant litter size x LPS interaction; F_(1,19)_ = 9.46, *p* = 0.006; n = 5–7; Fig. 1C,E). Here LPS also led to a significant increase in CL plasma corticosterone that peaked at 60 min and had plateaued or was returning towards baseline by 90 and 120 min. In SL rats, the corticosterone response to LPS was slower and remained elevated at 120 min (litter size × LPS × time interaction; F_(4,128)_ = 7.46, *p* < 0.001; n = 6–13; Fig. 1D).

### LPS effects on hippocampal GR expression in neonatally overfed rats

**Figure 2:**
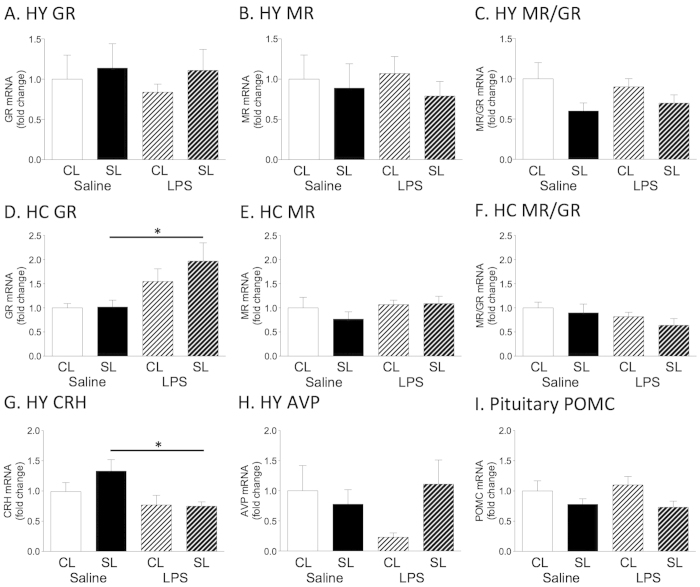
Hypothalamic (**A**–**C**) and hippocampal (**D**–**F**) expression of (**A**,**D**) glucocorticoid receptor (GR), (**B**,**E**) mineralocorticoid receptor (MR), (**C**,**F**) MR/GR ratio (**G**) hypothalamic expression of corticotropin-releasing hormone (CRH), (**H**) hypothalamic expression of arginine vasopressin (AVP), (**I**) pituitary expression of pro-opiomelanocortin (POMC) of adult rats raised in control (CL) and small (SL) litters 2 hr after i.p. LPS. Data are mean + SEM. *p < 0.05.

These exacerbated responses to LPS in neonatally overfed rats led us to examine what changes in the HPA axis may be responsible for these differences. When the HPA axis is activated, GC are released and these feed back onto GR in the hypothalamus and hippocampus to suppress further activity. We thus examined hypothalamic and hippocampal GR and mineralocorticoid (MR) expression under basal conditions and after an LPS challenge to assess the capacity for GC negative feedback in SL and CL rats (n = 7–8). In the hypothalamus there was no effect of litter size or LPS on GR or MR expression and no effect when GR mRNA was expressed as a ratio to MR (Fig. 2A–C). In the hippocampus, there was a significant effect of LPS (F_(1,26)_ = 10.11, *p* = 0.004), with LPS increasing GR expression in SL but not CL (Fig. 2D). LPS and neonatal overfeeding did not affect hippocampal MR or MR/GR ratios (Fig. 2E,F).

### LPS effects on hypothalamic corticotropin-releasing hormone in neonatally overfed rats

We next examined potential changes in the expression of various genes known to regulate HPA axis function at the level of the hypothalamus and pituitary (n = 7–8 as above). In these experiments SL rats had reduced corticotropin-releasing hormone (CRH) mRNA after LPS compared with saline (significant effect of LPS (F_(1,29)_ = 7.34, *p* = 0.012; Fig. 2G). Arginine vasopressin (AVP) mRNA levels were similar between all the groups (Fig. 2H). There was a significant effect of litter size on pituitary pro-opiomelanocortin (POMC) expression (F_(1,28)_ = 5.35, *p* = 0.028), with SL having reduced POMC expression compared with CL, but there were no differences with *post hoc* tests (Fig. 2I).

### The LPS-induced increase in melanocortin 2 receptor adrenal gene expression is suppressed in neonatally overfed rats

**Figure 3:**
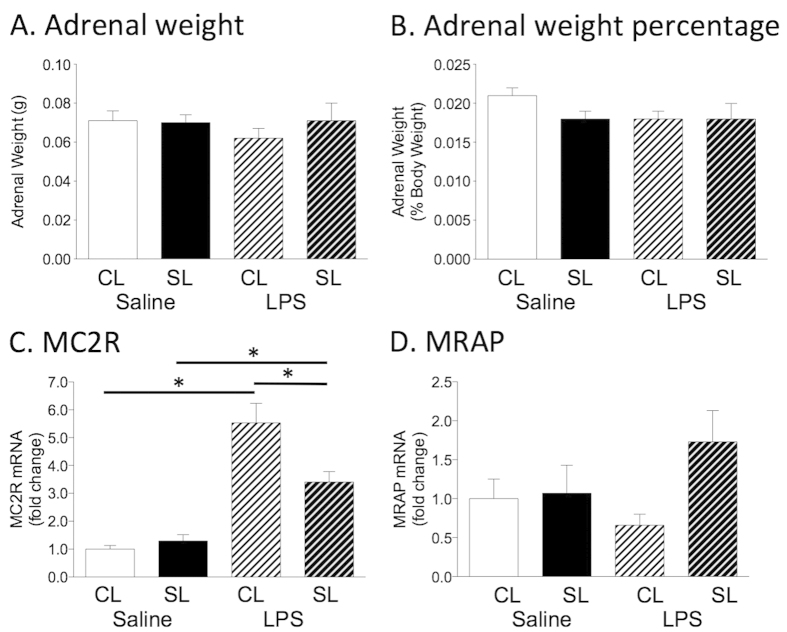
(**A**) Absolute adrenal weight, (**B**) adrenal weight expressed as a percentage of total body weight, (**C**) adrenal gland expression of melanocortin 2 receptor (MC2R) mRNA, (**D**) adrenal expression of melanocortin receptor accessory protein (MRAP) mRNA of adult rats raised in control (CL) and small (SL) litters 2 hr after i.p. LPS. Data are mean + SEM. *p < 0.05.

When the HPA axis is stimulated, adrenocorticotropic hormone (ACTH) released from the anterior pituitary acts at the melanocortin 2 receptor (MC2R) on the adrenal cortex to stimulate GC production and release. We therefore next examined adrenal expression of MC2R and its accessory protein, melanocortin receptor accessory protein (MRAP; n = 7–8 as above). There were no significant effects on absolute or percentage adrenal weights (Fig. 3A, B). LPS significantly elevated adrenal MC2R mRNA by 2 hr after injection in CL and SL rats (litter size x LPS interaction; F_(1,26)_ = 8.94, *p* = 0.006), but this effect was significantly less robust in the SL group compared with CL (Fig. 3C). We saw no significant differences in MRAP gene expression between the groups but the main effect of litter size on gene expression was *p* = 0.072 (Fig. 3D).

### Neonatally overfed rats have impaired *in vitro* release and inefficient *in vivo* suppression of ACTH-stimulated corticosterone

**Figure 4:**
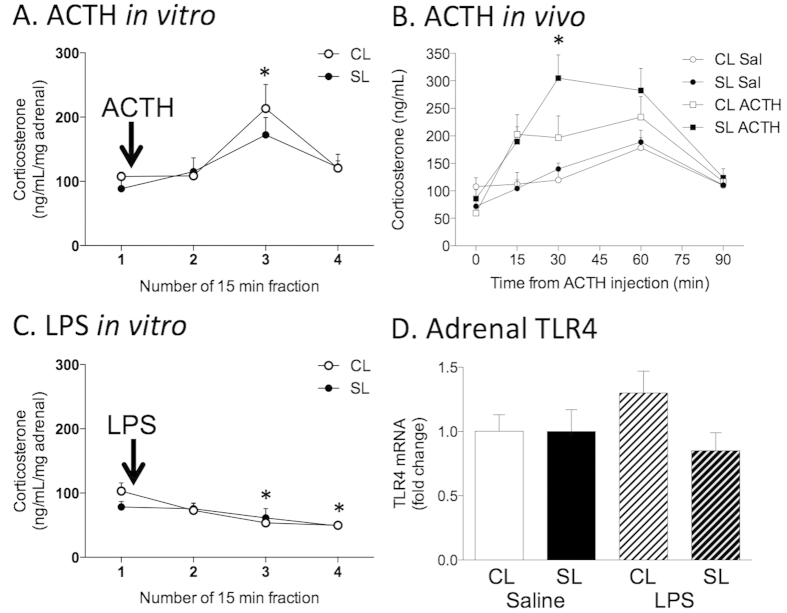
(**A**) Adrenocorticotropic hormone (ACTH)-induced corticosterone in adrenals from adult rats raised in control (CL) and small (SL) litters. ACTH (10^−7^ M added after collection of fraction 1). (**B**) ACTH-induced plasma corticosterone in adult CL and SL rats. (**C**) Lipopolysaccharide (LPS)-induced adrenal corticosterone. LPS (1 μg/mL added after collection of fraction 1). (**D**) Adrenal gland toll-like receptor (TLR)4 mRNA. Data are mean + SEM. (**A**) *compared with fraction 1, 2, and 4 for CL; *p* < 0.05, corrected for adrenal weight. (**B**) *compared with saline-treated group for the same litter size; *p* < 0.05. (**C**) *compared with fraction 1 for CL; *p* < 0.05, corrected for adrenal weight.

Our findings outlined above were indicating neonatally overfed rats had differences in the ability of the adrenal cortex to respond to the ACTH generated as part of the HPA axis response to LPS. To investigate if this was the case, we measured the *in vitro* effect of ACTH on the adrenal release of corticosterone. *In vitro,* ACTH stimulated significant adrenal corticosterone release in CL but not SL rats (significant effect of ACTH; F_(3,24)_ = 9.70, *p* < 0.001; Fig. 4A; n = 6 per group), with a significant difference seen 30 min after exposure to ACTH. These findings indicated neonatal overfeeding leaves the rats less able to respond to stimulation of the HPA axis, likely due to their reduced ability to increase expression of the MC2R.

To test if this was the case *in vivo*, we also measured plasma corticosterone responses to ACTH injection in CL and SL rats. In this experiment we found the corticosterone response to ACTH was small and not significantly different from the response to saline in CL rats. In SL, corticosterone was significantly increased relative to saline at 30 min after ACTH injection, indicative of a robust adrenal response in these rats (significant time × ACTH interaction; F_(4,128)_ = 7.93, *p* < 0.001; time × ACTH interaction; F_(4,128)_ = 2.31, *p* < 0.061; n = 9 per group; Fig. 4B).

To test if the differences between the *in vivo* responses to LPS and to ACTH were due to a direct effect of LPS at the adrenal gland we also stimulated adrenals *in vitro* with LPS and examined corticosterone responses. *In vitro* LPS mildly suppressed adrenal corticosterone release in CL (significant effect of ACTH; F_(3,24)_ = 5.61, *p* = 0.005; Fig. 4C; n = 6 per group), with significant differences from baseline seen at 30 and 45 min after LPS exposure, but there were no effects in SL adrenals. Neonatal overfeeding also did not alter adrenal expression of TLR4 (Fig. 4D).

## Discussion

Early life events have established and pronounced effects on HPA axis function. Here we present the first evidence that the adrenal MC2R may be involved in the response, with early life overfeeding altering the LPS-induced expression of this receptor. Thus, as we have previously reported, male rats made overweight long-term by early life overfeeding have exacerbated central (hypothalamic) neuronal activation in response to LPS^4^,^5^. In a similar profile to our previous findings, the GC response in the neonatally overfed is slower, with corticosterone taking longer to reach a peak and return to baseline^4^. In accordance with this finding, expression of the MC2R gene is significantly and substantially elevated after LPS in rats fed a control diet as neonates, but in the neonatally overfed this response is comparatively suppressed.

Upon exposure to LPS, cytokines such as interleukin (IL)-1, tumor necrosis factor (TNF)α and IL-6, stimulate activation of the PVN leading to downstream ACTH release from the pituitary, which interacts with MC2R and leads to GC release from the adrenal. Indeed, in neonatally overfed animals, the cytokine response to LPS is exacerbated, likely leading to enhanced stimulation of the HPA axis at its apex^4^. LPS-stimulated GC normally inhibit further pro-inflammatory cytokine transcription and feed back directly and indirectly onto the PVN to inhibit further HPA axis activation and GC release^21^,^22^. Our observation that the GC response to LPS is slower and the PVN response exacerbated in SL rats led us to hypothesise GC negative feedback would be less efficient at the level of the hypothalamus or hippocampus as has been previously seen with models of maternal care^9^,^10^,^11^,^12^. Unlike in rats given low levels of maternal care as neonates, whose hippocampal GR expression is suppressed leading to exacerbated HPA axis responses to stress, neonatal overfeeding induced no such changes in GR expression. Although hippocampal GR expression was significantly increased in SL rats and not CL after LPS, there was no significant difference between the CL and SL groups in this response. There was also no difference between the groups in MR expression or the MR:GR ratio, important for assessment of MR:GR balance as an indicator of HPA axis function^23^,^24^. It is therefore unlikely neonatal overfeeding is influencing the HPA axis response to LPS by altering negative feedback at the hypothalamus and hippocampus. It is noteworthy that these early studies by Meaney and colleagues describe an excellent relationship between GR gene and protein expression^25^, as does our previous study with early life immune challenge^16^. As we saw no differences between the CL and SL groups in gene expression, we did not follow up with assessment of the protein. As with GC negative feedback, the ability of the PVN CRH cells to respond to LPS is probably also intact in that neonatal overfeeding had no effect on CRH or AVP expression either under basal conditions or after LPS, except that LPS suppressed CRH expression in SL but not significantly in CL. Pituitary POMC expression was likewise not different between the groups, indicating the ability to produce ACTH in response to a stimulus is probably not affected by neonatal diet.

Neonatal diet did, however, significantly affect adrenal MC2R expression in response to LPS. It would be very interesting to examine if MC2R protein levels reflect the changes in gene expression we see. However, we could not eliminate non-specific bands with the commercially available antibodies (data not shown). Others have also reported that there is currently no suitable antibody for this protein^26^. This change in MC2R gene expression at least implies the SL adrenal may be less able to efficiently respond to LPS-induced ACTH to stimulate GC release. From this finding, we hypothesized that the GC response to ACTH alone would also be attenuated in SL rats and this was indeed the case in our *in vitro* preparation. ACTH administrated *in vivo* to neonatally overfed rats actually led to more GC release at 30 min after injection in these than in controls, potentially reflecting differences in the time course of the response to LPS versus ACTH alone or implying LPS might act directly at the adrenal to dampen GC release.

Although there is little published data on the adrenal MC2R and how LPS may affect it, there is some suggestion that LPS may directly influence the MC2R-mediated GC response. LPS can suppress the ACTH-induced GC response in fasciculata reticularis and glomerulosa cells^27^,^28^. Prior exposure to LPS can induce endotoxin tolerance in the adrenal, with the corticosterone response to LPS or ACTH being suppressed in primary fasciculata reticularis cells from rats that had previously been given LPS^29^. Importantly, MC2R-/- mice do not mount a GC response to LPS (or to restraint stress), indicating that MC2R is necessary for both the ACTH-mediated and direct LPS-mediated GC response^30^. The MC2R is a G protein-coupled receptor whose activation is dependent upon the presence of the accessory protein MRAP. The activation of MC2R leads to conversion of ATP into cAMP via stimulation of adenylyl cyclase. Activation of protein kinase A ensues and leads to phosphorylation of cAMP response element protein, activating the transcription of steroidogenic acute regulatory protein (StAR) and other genes involved in steroidogenesis^31^,^32^. There is some evidence LPS can interact with this mechanism, modifying the binding of ACTH to the cell membrane and modifying the ACTH signal transduction pathway, suppressing ACTH-induced cAMP production in primary culture^27^. However, it is unlikely the differences we see between controls and neonatally overfed are due to a direct action of LPS at the level of the adrenal. Expression of adrenal TLR4 was not affected by litter size. Additionally, while LPS led to a suppression of corticosterone after 30 and 45 min in CL in our *in vitro* preparation, no such effects were seen in SL.

**Figure 5:**
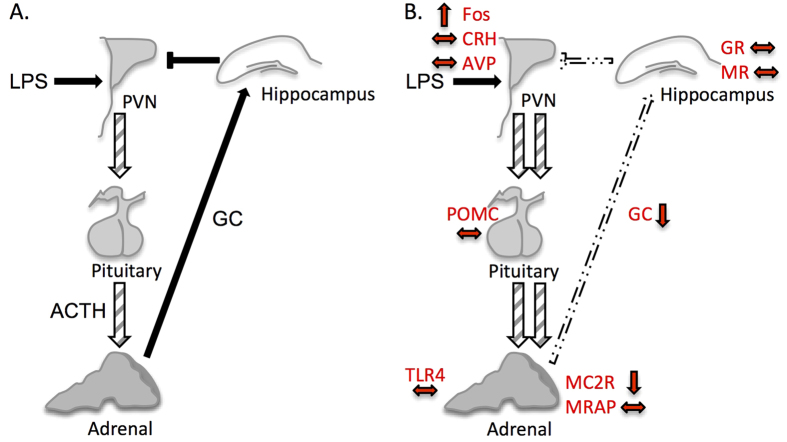
Proposed circuitry by which neonatal overfeeding leads to exacerbated hypothalamic-pituitary-adrenal (HPA) axis responses to lipopolysaccharide (LPS). (**A**) Under normal conditions LPS acts at the level of the brain to stimulate HPA axis activation and glucocorticoid (GC) production. GC feed back centrally to dampen further HPA axis activation. (**B**) In neonatally overfed rats, central HPA axis responses to LPS are likely to be normal, stimulating paraventricular nucleus of the hypothalamus (PVN) activation and adrenocorticotropic hormone (ACTH) release from the pituitary. The ability of GC to suppress further HPA axis activity at the level of the brain is also normal. However, the effect of ACTH on the adrenal is impaired leading to slower LPS-induced activation of MC2R-mediated GC release, slower GC negative feedback and exaggerated PVN neuronal activation. Red arrows indicate direction of gene differences between CL and SL groups after LPS treatment. AVP, arginine vasopressin; CRH, corticotropin releasing hormone; GC, glucocorticoids; GR, glucocorticoid receptor; MC2R, melanocortin 2 receptor; MR, mineralocorticoid receptor; MRAP, melanocortin receptor accessory protein; POMC, pro-opiomelanocortin.

Collectively, our data strongly suggest the central HPA axis response to LPS in neonatally overfed rats is probably normal, but that there is a change in the sensitivity of the adrenal gland to the ACTH released after HPA axis activation such that its direct effects are modified (Fig. 5). Thus, neonatal overfeeding leads to a suppression of the MC2R-mediated response to ACTH and a suppressed increase in expression of MC2R. In the *in vivo* presence of LPS this is seen as slower GC release and thus slower GC negative feedback to suppress the response. In the case of a single stimulus with ACTH, it is likely the GC response is already nearly resolved by 15 min in the CL^33^, but the SL response is again impaired or less efficient. The differences in the time courses of these stimuli (LPS and ACTH alone) may explain the differences in the profiles of the corticosterone release. It is noteworthy in this regard that non-genomic intra-adrenal negative feedback to suppress ACTH-mediated GC release has recently been identified and this acts very rapidly, i.e. within minutes^34^. The exact molecular mechanisms behind the changes we see remain to be determined, but the implication of these data and that they have exacerbated febrile and cytokine profiles after LPS^4^, is that neonatally overfed animals have a less efficient adrenally-mediated response to bacterial endotoxin. In this regard, neonatally overfed rats may therefore be less able to combat bacterial infection. There is also the possibility that this inefficiency could be compensated for with supplementary GC, an idea that also requires further study.

## Methods

### Animals

We obtained timed pregnant Wistar rats from the Animal Resources Centre, WA, Australia. They were maintained at 22 °C on a 12 hr light / dark cycle (0700–1900 hr) with pelleted rat chow and water available *ad libitum*. All procedures were conducted in accordance with the National Health and Medical Research Council Australia Code of Practice for the Care of Experimental Animals and were approved by the RMIT Animal Ethics Committee.

### Litter manipulation

On the day of birth (P0) we removed all the pups from their dams and randomly reallocated them to new dams in litters of 4 or 12 as we have described previously^18^,^19^,^20^. Care was taken that no dam received any of her own pups. Each new litter was made up of 50% males and 50% females. Excess pups were culled. Females were used in other experiments.

Following pup reallocation, the litters were weighed weekly as whole litter units; we have previously shown that males and females show similar growth rates until after weaning^35^. At weaning the pups were separated into same-sex littermate pairs where they were left undisturbed, except for the usual animal husbandry, until experimentation. All experimental groups were derived from 3 or more litters, using a maximum of 2 pups from the same litter for an experimental treatment. N are indicated in the results.

### HPA axis responses to immune challenge with i.p. LPS

At approximately P70, we took each rat from its cage and nicked the end off the tail with a sharp razor blade to extract a ~20 μL baseline blood sample into a heparinized capillary tube. We collected each sample within 3 min of nicking the tail to minimize any handling effects on the corticosterone levels detected in the sample^36^. We then gave each rat an i.p. injection of LPS (*Escherichia coli*, serotype 026:B6; L-3755; Sigma, St Louis, MO, USA; 100 μg/kg in 1 mg/kg pyrogen-free saline), or pyrogen-free saline and took blood samples at 30, 60, 90, and 120 min after injection. Blood samples were kept on ice until the end of the experiment, when they were centrifuged and the plasma aliquots stored at −20 °C until assayed.

At 120 min after injection, and immediately after the final blood sample, we deeply anaesthetized the rats with Lethabarb (~150 mg/kg pentobarbitone sodium, i.p.) and perfused them transcardially with phosphate buffered saline (PBS; 4 °C, pH 7.4) followed by 4% paraformaldehyde in PBS (4 °C, pH 7.4) for collection of fixed brains. We then removed the brains and post-fixed them for 4 hr in the same fixative before placing them in cryoprotectant with 20% sucrose in PBS (4 °C). We subsequently cut forebrains into 40 μm coronal sections using a cryostat. All experiments were initiated between 0900 and 1200 hr to limit potential effects of circadian rhythms on any parameters measured.

### Fos immunohistochemistry

We assessed neuronal activation in the fixed brains on the basis of positive Fos-immunoreactivity, seen as a black deposit in the nucleus. Briefly, a one-in-four series of forebrain sections from each animal was incubated in primary Fos antibody (overnight; 4 °C; 1:10 000; rabbit polyclonal; Santa Cruz Biotechnology, Santa Cruz, CA, USA), then in secondary antibody (90 min; room temperature; 1:200; biotinylated anti-rabbit; Vector Laboratories, Burlingame, CA, USA) and in an avidin-biotin horseradish peroxidase (HRP) complex (1 hr; Vector Elite kit, Vector). The sections were then incubated in nickel diaminobenzidine to visualize the HRP activity, seen as a black nuclear deposit. The reaction was terminated once an optimal contrast between specific cellular and non-specific background labelling was reached. Sections from each treatment group were processed simultaneously. Sections were mounted on chrome-alum-coated slides, dehydrated in a series of alcohols, cleared in Histoclear and coverslipped.

### Cell counts

An experimenter, blinded to the group treatments, carried out counts of cells positive for Fos-immunoreactivity in the PVN. These were counted over two sections (~1.8 and 1.96 mm caudal to bregma).

### Corticosterone assays

We measured plasma corticosterone concentrations using a standard rat corticosterone ELISA (Abnova Corp., Taipei, Taiwan). The inter-assay variability for this assay was 7.2% coefficient of variation (CV), intra-assay variability 4.8% CV, and lower limit of detection 40 pg/mL. We assayed samples from all treatment groups together in duplicate.

### Real time reverse transcriptase polymerase chain reaction (rt-PCR) analysis

To determine candidate steps in the HPA axis that were altered by neonatal overfeeding, we also took a cohort of CL and SL male rats at P70 and collected fresh tissue for rt-PCR at 2 hr after LPS (or saline) injection. We deeply anaesthetised the rats with Lethabarb and quickly removed hypothalamus, hippocampus, pituitary, and adrenal glands over ice. We weighed adrenals, immediately snap-froze all tissues in liquid nitrogen, and stored them at −80 °C until use.

We isolated RNA from brain, pituitary, and adrenals using QIAzol and an RNeasy purification kit (QIAGEN, Valencia, CA, USA). RNA (1 μg) was transcribed to cDNA using an iScript cDNA synthesis kit (Bio-Rad Laboratories, Hercules, CA, USA), following the manufacturers’ instructions. Real-time rt-PCR was performed using Taqman Gene Expression Assays (Table 1; Applied Biosystems, Mulgrave, Vic, Au). Fold differences in target mRNA expression were measured using the delta-cycle threshold method by comparison with the housekeeping gene, 18S^37^,^38^ and expressed as mRNA relative fold change as described previously^16^,^39^.

### Corticosterone responses to adrenocorticotropic hormone

As our data suggested the effect of neonatal overfeeding on HPA axis function was principally due to changes in the adrenal gland, we examined corticosterone responses to stimulation of the adrenal *in vivo* with ACTH (1.5 μg/kg in 1 mL/kg pyrogen-free saline, s.c). We gave CL and SL adult rats ACTH and took blood samples immediately prior to injection and 15, 30, 60, and 90 min after injection. Samples were analysed as above.

### Adrenal corticosterone responses *in vitro*

To assess the *in vitro* effect of ACTH and LPS on the adrenal release of corticosterone, we deeply anaesthetised CL and SL rats with Lethabarb, excised their adrenal glands and stored the adrenals in ice-cold Dulbecco’s modified Eagle’s medium/Nutrient mixture F-12 (DMEM/F-12; Thermo Fisher Scientific, Scoresby, Victoria, Australia) containing 0.1% BSA until all tissues were collected. We then bisected each adrenal gland, weighed and pre-incubated each half adrenal gland for two × 1 hr in 1 mL of DMEM/F-12 at 37 °C in a 95% O_2_/5% CO_2_ atmosphere. After the pre-incubation period, we refreshed the medium and collected samples every 15 min. ACTH (10^−7^ M)- and LPS (1 μg/mL)-containing medium was added in the second fraction. At the end of each 15 min period the medium was collected and stored in −20 °C until assayed for corticosterone levels using a standard rat corticosterone ELISA, as described above. The protocol was adapted from^40^,^41^,^42^ and we determined doses in pilot experiments.

### Data analysis

We compared pre-weaning body weights between CL and SL rats using an analysis of variance (ANOVA) with repeated measures, with litter size as the between factor and age as the repeated measure. When a significant interaction was found between litter size and age, we performed Student’s unpaired t-tests for each time point. We compared adult weights, Fos-positive cell counts and gene expression using two-way ANOVAs with litter size and LPS-treatment as between factors. Corticosterone concentrations were compared using repeated measures ANOVAs with litter size and LPS- or ACTH-treatment as the between factors and time as the repeated measure. We used Tukey *post hoc* comparisons where significant interactions were found. Data are presented as the mean ± standard error of the mean (SEM). Statistical significance was assumed when *P* < 0.05.

## Additional Information

**How to cite this article**: Cai, G. *et al.* Overfeeding during a critical postnatal period exacerbates hypothalamic-pituitary-adrenal axis responses to immune challenge: a role for adrenal melanocortin 2 receptors. *Sci. Rep.* **6**, 21097; doi: 10.1038/srep21097 (2016).

## Figures and Tables

**Table 1:** TaqMan probe details (Life Technologies) used for qRT-PCR.

Target Gene	NCBI Reference Sequence	TaqMan Assay ID	Product Size
*Nr3c1*	NM_012576.2	Rn00561369_m1	73
*Nr3c2*	NM_013131.1	Rn00565562_m1	79
*Crh*	NM_031019.1	Rn01462137_m1	112
*Avp*	NM_016992.2	Rn00690189_g1	78
*Pomc*	NM_139326.2	Rn00595020_m1	92
*Mc2r*	NM_001100491.1	Rn02082290_s1	126
*Mrap*	NM_001135834.1	Rn01477212_m1	62
*Tlr4*	NM_019178.1	Rn00569848_m1	127
*18s*	X03205.1	4319413E	187
